# Numerical Study of the Simultaneous Oxidation of NO and SO_2_ by Ozone

**DOI:** 10.3390/ijerph120201595

**Published:** 2015-01-29

**Authors:** Bo Li, Jinyang Zhao, Junfu Lu

**Affiliations:** 1 Electric Power Planning & Engineering Institute, Ande Rode No. 65, Xicheng District, Beijing 100120, China; E-Mail: jyzhao@cpecc.net; 2 Key Laboratory for Thermal Science and Power Engineering of Ministry of Education, Department of Thermal Engineering, Tsinghua University, Beijing 100083, China; E-Mail: lvjf@mail.tsinghua.edu.cn

**Keywords:** ozone injection, kinetic modeling, nitrogen oxides, sulfur dioxide, multi-pollutant control technology

## Abstract

This study used two kinetic mechanisms to evaluate the oxidation processes of NO and SO_2_ by ozone. The performance of the two models was assessed by comparisons with experimental results from previous studies. The first kinetic mechanism was a combined model developed by the author that consisted of 50 species and 172 reactions. The second mechanism consisted of 23 species and 63 reactions. Simulation results of both of the two models show under predictions compared with experimental data. The results showed that the optimized reaction temperature for NO with O_3_ ranged from 100~200 °C. At higher temperatures, O_3_ decomposed to O_2_ and O, which resulted in a decrease of the NO conversion rate. When the mole ratio of O_3_/NO was greater than 1, products with a higher oxidation state (such as NO_3_, N_2_O_5_) were formed. The reactions between O_3_ and SO_2_ were weak; as such, it was difficult for O_3 _to oxidize SO_2_.

## 1. Introduction

Air pollution is now one of the most serious environmental problems worldwide. The flue gas of coal-fired power plants usually contains fine particles such as SO_2_, NO_X_ and mercury [1,2]. Although China is a country with many coal-fired power plants, emission standards have been strengthened in recent years [3]. Pollution control systems are required in order to meet these newer strict emission regulations. Conventional coal-fired power plants usually combine multiple single pollutant control facilities for emission control purposes. In recent years, various kinds of technologies have been developed to reduce worldwide SO_2_ and NOx emissions [4,5,6]. In order to remove SO_2_, several wet desulphurization technologies, including the calcium-gypsum process, magnesium oxide scrubbing and the double alkaline process have been widely applied in coal-fired power plants. Typically, one of these three methods is adopted to achieve NOx emission control [7,8]. These methods include Low-NOx burner technology (used to reduce the formation of NOx in the furnace), Selective Catalytic Reduction (SCR) technology and Selective Non-Catalytic Reduction technology (SNCR).

A multi-pollutant control system is defined as: a system that can remove two or more of the principle regulated pollutants (SO_2_, NOx particulate matter, mercury and CO_2_) in a single reactor or a single system designed for control purposes [9]. Numerous simultaneous removal techniques have been previously investigated [10,11], including the dry absorption method [12], the electron beam process [13] and the wet oxidation method [14]. In the wet oxidation method, various oxidation agents are used to oxidize NOx and SO_2_, including chlorine dioxide [15], sodium chlorite [16], hydrogen peroxide [17] and ozone [18,19]. As a stable molecule, O_3_ is a potentially useful oxidant in practical applications. O_3_ is generated in a non-thermal plasma reactor and then injected into the flue gas duct or a NOx reactor. NO_X_ and SO_2_ are then oxidized into higher oxides and mercury is able to be oxidized into mercury oxide. Numerous studies have investigated the O_3_ oxidation of flue gas [20,21,22,23,24,25,26]. These studies demonstrated that ozone could be an efficient oxidizing agent for the oxidation of NO. Wang* et al.* experimentally investigated the simultaneous removal of NOx and SO_2_ in the nitrogen flow of a narrow reactor by ozone injection [20]. The results showed that the injection of ozone could simultaneously react with both NO and SO_2_, with NO being oxidized to a higher oxidation state. Also, in this study, the optimal temperature for NO oxidation of 473 K was suggested. Sun* et al.* studied the O_3_ oxidation processes of NO and SO_2_ using an* in situ* IR spectrometer [21]. Experimental results showed that the O_3_ concentration and the reaction temperature played critical roles in the O_3_ oxidation process of NO. When the molar ratio of O_3_/NO was greater than 1, the oxidation products were NO_2_, N_2_O_5_ and HNO_3_; however, O_3 _did not significantly oxidized SO_2_ for that this reaction has high energy barrier. Skalska* et al.* investigated the intensification of the NOx absorption process by means of ozone injection into the exhaust gas stream; results showed that, due to oxidation, the efficiency of the NOx absorption was much higher than without the ozone injection [22], the reason is that NO could be efficiently oxidized by O_3_ into NO_2 _and other products of higher oxidation state of NO, these products are water-soluble and can be absorbed by alkaline solution. Mok proposed a two-stage process consisting of an ozonizing chamber and an absorber containing a reducing agent solution. The injection of ozone led to rapid oxidation of NO to a higher oxidation state and NOx removal was achieved by absorption using sodium sulfide along with the removing of SO_2_ [23]. Sun* et al.* studied the simultaneous absorption process of NOx and SO_2_ from flue gas with a pyrolusite slurry combined with a gas-phase oxidation of NO using ozone [24]. The results revealed that ozone could oxidized NO to NO_2_ with a high degree of efficiency. The MnO_2_ in the pyrolusite slurry oxidized SO_2_ and NO_2_ into MnSO_4_ and Mn(NO_3_)_2_, respectively, in the liquid phase.

Due to a lack of kinetic models, previous studies rarely provided a detailed chemical process of NO and SO_2_ oxidization by O_3_. As such, the interactions of NO and SO_2_ during the oxidation process have not been fully investigated. This paper focuses on the chemical process in its presentation of a detailed study on the kinetic mechanisms of the oxidation of NO and SO_2_ by O_3_. Based on the findings, the optimized reaction conditions for the practical application of O_3_ injections for multi-pollutant controls in coal-fired power plants are provided. In particular, this study considers two mechanisms in order to evaluate the oxidation process. The performance of the two mechanisms are first assessed by comparisons with experimental data from Mok* et al.* [25] and Stamate* et al.* [26]. The oxidation of NO and SO_2_ by O_3_ are then discussed by comparisons of simulation results with experimental results from Wang* et al.* [27] (in Chinese) and Wang* et al.* [28] (in Chinese). Next, further insights are provided into the kinetic mechanisms that control the oxidation process of NO and SO_2_ with O_3_.

## 2. Experimental Setup

**Figure 1:**
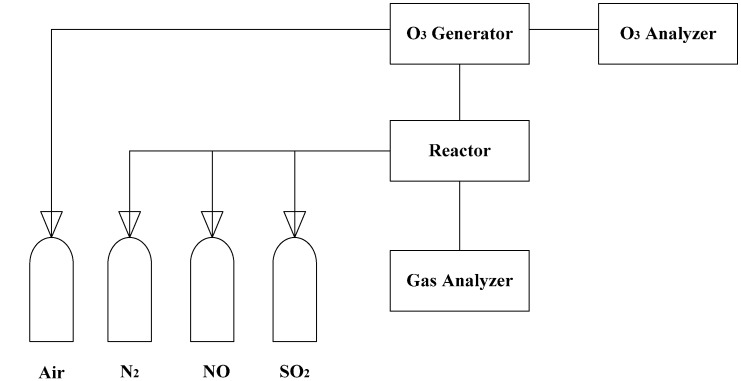
Schematic diagram of the experimental apparatus [20].

A multi-layers plug-flow quartz reactor was used for the experiment setup [20]. The experiment apparatus consisted of ozone generation, a quartz flow reactor, an O_3_ analysis system and an online gas analysis system (Figure 1).

**Figure 2:**
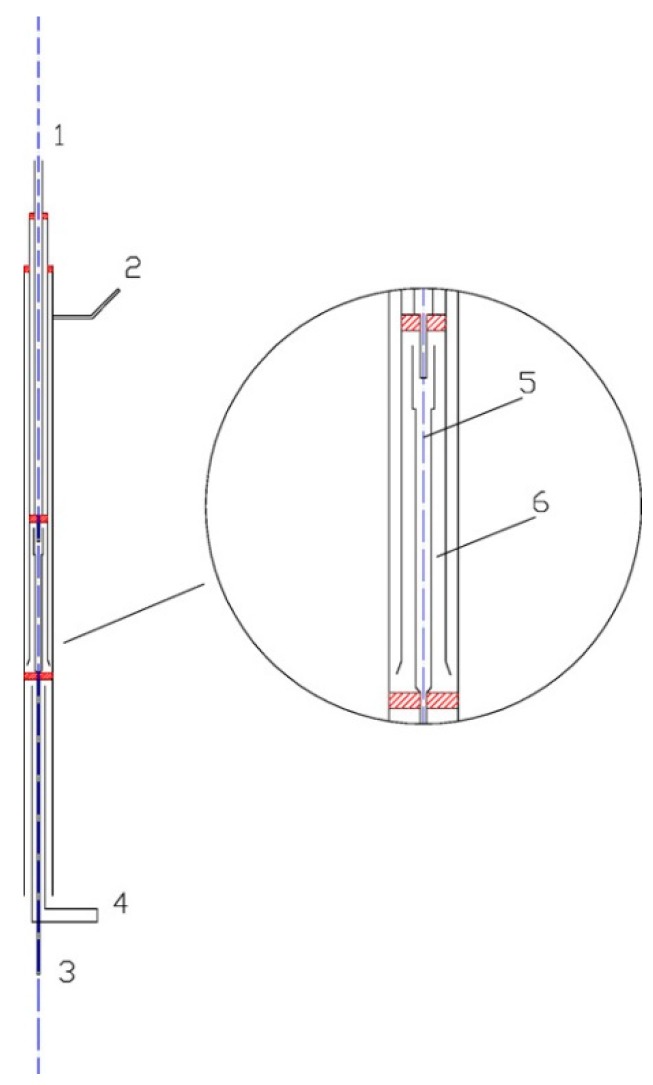
Schematic of the quartz flow reactor. (1) Inlet 1; (2) Inlet 2; (3) Outlet; (4) Air cooling; (5) Quartz flow reactor; (6) Preheat channels [20].

The oxidation reactions occurred in the quartz flow reactor, as shown in Figure 2. A special delicate flow reactor (with a three-channels homocentric reaction tube) was designed and made by quartz glass in order to reduce the impact of the non-uniform temperature profile of the furnace. The reactor had a total length of 642 mm and an outer-diameter of 20 mm. The heating length of the electric furnace was 600 mm. The reactions occurred in the center tube. The tube had an inside diameter of 5 mm and a length of 100 mm. The center tube was surrounded by O_3_, and the temperatures of O_3 _and the mixtures in the center tube were identical, thus it could be considered that there was no heat transfer between the center tube and outside. Therefore, the center tube was treated as an isothermal reactor. The NO/SO_2_/N_2_ mixtures entered the reactor through inlet 2 and then passed through a reciprocating preheating channel before entering the center tube. O_3_ was generated in the ozone generator; it then flowed into the reactor through inlet 1 where it was heated by a separate preheating channel. The two well-preheated streams instantly mixed with each other at the nozzle of center tube.

The ozone was generated by a dielectric barrier discharge (DBD) device with 3.7–4 kVAC voltage and 5 kHz (model CF-G-3-010G). The output concentration of O_3_ was continuously monitored by an ozone analyzer. The simulated flue gas was prepared by N_2_ and small amount of concentrated NO gas and SO_2_ gas. Oxygen was not absent in the main flow in these experiments. The flow rate of the gas was all controlled by mass flow controller [20]. The total flow rate of all reactants, including O_3_ and simulated flue gas, were fixed to 1000 mL/min. The residence time in the center tube ranged from 0.049‒0.089 s and varied with temperature (calculated by 33.3 K/T s).

## 3. Kinetic Modeling and Numerical Simulation Methodology

### 3.1. Kinetic Mechanism Description

This study used two kinetic mechanisms to model the oxidation process of NO/O_3_, SO_2_/O_3_ and NO/SO_2_/O_3_. The first kinetic mechanism is a combined model developed by the author, consisting of 50 species and 172 reactions (Model I). Model I considered three kinds of reactions: NO/O_3_ reactions (referenced in Wen* et al.* [29]); SO_2_/O_3_ reactions (referenced in Wang [28]); and NO/SO_2_ reactions (referenced online [30]). The second kinetic mechanism was developed by Sun* et al.* [21] based on the situ IR spectrometer measurements. It consisted of 23 species and 63 reactions (Model II). Table 1 shows the key reactions and parameters for Model I and Model II. The key reactions of the two models were identical and the parameters were different. The supplementary file lists the other reactions of the two models. Most of the reactions of the two models were different. For example, there were 112 reactions to describe the oxidation process of SO_2_ and SO_2_/NO in Model I and 8 reactions for Model II.

**Table 1:** Comparisons of parameters for key reactions of Model I and Model II.

Reactions	Model I		Model II	
	A (cm^3^/mol-s)	Ea (cal/mol)	A (cm3/mol-s)	Ea (cal/mol)
O_3_ + NO = NO_2_ + O_2_ (R1)	1.8E + 12	2722	8.43E + 11	2605
O_3_ + NO_2 _= O_2_ + NO_3 _(R2)	7.22E + 10	4870	8.43E + 10	4913
O_3_ + SO_2_ = O_2_ + SO_3 _(R3)	1.81E + 12	13,910	1.81E + 12	13,923
O_3_ = O_2_ + O (R4)	2.0E + 15	23,250	4.31E + 14	22,277
O_3_ + O = O_2_ + O_2_ (R5)	4.82E + 12	4093	4.82E + 12	4098

Model I is a detailed mechanism in which the three kinds of reactions are validated by experimentation [28,29,30]. Model II is relatively small and can reduce computational costs when used for predictions. The differences in the reaction parameters may be the result of differences in the reaction paths and, therefore, the prediction results. The following sections address this matter in further detail.

### 3.2. Simulation Strategy

The present kinetic calculations were performed using the Plug Flow Reactor (PFR) computer code [31] which was used to predict the oxidation process of NO/O_3_, SO_2_/O_3_ and NO/SO_2_/O_3_ in a multi-layers plug-flow quartz reactor. The PFR model was used to describe a suitable steady-state, tube flow reactor for process design, optimization and control. The mixing in the axial flow direction was ignored, but perfect mixing in the directions transverse to this were taken into account for the PFR models. Thermodynamic curve fits were obtained from the National Institute of Standards and Technology chemical species database [32].

The gaseous mixtures were introduced into the PFR at 101.3 kPa. The starting point of the distance was x = 0. In Section 4.1, the initial NO concentration, the molar ratio of NO/O_3_ and the temperature were maintained in accordance with experiments from Mok* et al.* [25] and Stamate* et al.* [26], detailed experimental conditions are introduced in Section 4.1. In Section 4.2 and Section 4.3, the diameter and the length of the PFR reactor were kept at 5 mm and 100 mm. The residence time were ranged from 0.049‒0.089 according to different temperatures. In Section 4.1, the initial NO concentration, the molar ratio of NO/O_3_ and the temperature were maintained in accordance with experiment [27]. Regard to the discussion of temperature effect on the NO conversion rate, the initial NO concentration was 300 ppm, O_3_/NO mole fraction was 1.0, the residence time was kept at 1 s. Regard to the discussion of the effect of the molar ratio of O_3_/NO on reaction products, the temperatures were kept at 100 °C and 200 °C respectively, the initial NO mole fraction was maintained at 300 ppm, O_3_/NO mole fraction was 1.5 and 2.0, the residence time was kept at 1s. In Section 4.3, the initial SO_2_ concentration, the molar ratio of SO_2_/O_3_ and the temperature were maintained in accordance with experiment [28]. In Section 4.4, the O_3_/NO/SO_2_ mole fraction was 2:1:1 and the initial concentration of NO and SO_2_ was 300 ppm.

## 4. Results and Discussion

### 4.1. Model Validation

**Figure 3:**
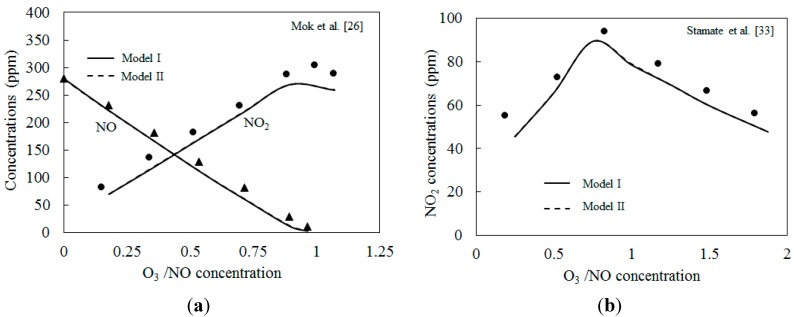
Comparison of predicted results with experimental data. (**a**) Initial NO: 280 ppm, NO_2_: 20 ppm, 25 °C; (**b**) Initial NO: 80 ppm, 40 °C.

The two kinetic models are first validated with the published experimental results. Figure 3 depicts the comparison of predicted results with the experimental data of NO_2_ concentration from Mok* et al.* [25] and Stamate* et al.* [26]. The experiments in Ref. [25] were conducted in an ozonizing chamber. O_3_ produced in a DBD device was continuously fed to the ozonizing chamber to convert NO into NO_2_. The exhaust gas was prepared by mixing air and small amounts of concentrated NO gas balanced with N_2_. The residence time was kept at 2.9 s. In Ref. [26], a 4.5 m long and 0.6 m in diameter reactor with a residence time of the flue gas of about 5 s was used to oxidize the NO. NO_2_ in the reactor were measured by FTIR. As shown in Figure 3, the numerical simulation results calculated by the two models were lower than those of the experimental results. The NO_2_ concentrations first increased and then decreased. Where the molar ratio O_3_/NO equaled 1.0, the concentration of NO_2_ reached a maximum value. This is in accordance with previous findings [33]. Skalska* et al.* investigated the mole concentration of products at different O_3_/NO mole fractions experimentally [33], results revealed that if the molar ratio of O_3_/NO is higher than 1.0, NO_2_ will react with O_3_ and generate higher nitrogen oxides, NO_3_. Also, N_2_O_5_ is formed as a result of the reaction of NO_2_ with NO_3_. The under-predictions of simulations shown in Figure 3a,b are probably caused by both the experimental uncertainties and the estimation uncertainties of reaction parameters in the reactions of the two kinetic models. More work is necessary to be conducted on the kinetic modeling in the future.

### 4.2. NO Oxidation by O_3_

**Figure 4:**
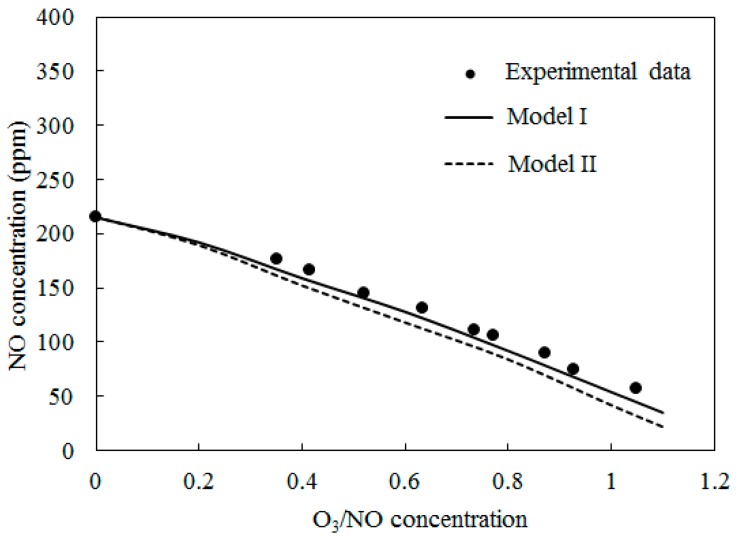
Variations of NO concentration with O_3_/NO concentrations at T = 100 °C.

Figure 4 depicts the concentrations of NO obtained by varying the O_3_/NO concentrations at temperature T = 100 °C. Here, the O_3_/NO concentrations ranged from 0.0 to 1.1. Figure 4 also provides comparisons of the experimental data [27] and simulation results with Model I and Model II. The NO concentration decreased as the O_3_/NO concentrations increased. Where the O_3_/NO concentration was less than 1.0, O_3_ reacted with NO via O_3_ + NO = NO_2_ + O_2_ (R1) and generated NO_2_. This conclusion is also confirmed by Figure 3a, as shown in Figure 3a, NO_2_ was first increased as NO decreased, which proved that R1 plays an important role in the process of NO oxidation. Higher nitrogen oxides were more difficult to form because there was not enough O_3_ to react with NO_2_. For O_3_/NO, the concentration was 1, the experiment determined the NO concentration as 56 ppm and the simulation results of Model I and Model II were 48 ppm and 25 ppm, respectively. Hence, at T = 100 °C, the effect of the O_3_ injection into NO was that O_3_ could provide stronger oxidization help to transform NO into a higher oxidation state.

**Figure 5:**
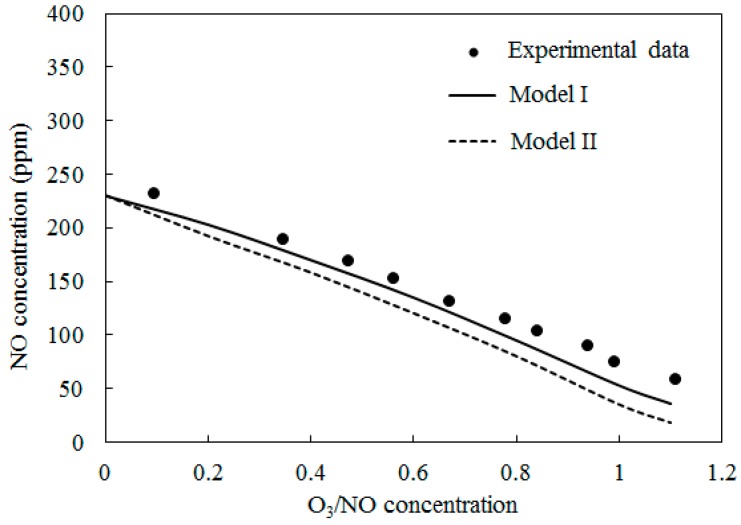
Variations of NO concentration with O_3_/NO concentrations at T = 200 °C.

Figure 5 depicts the comparisons of the computed NO concentration using Model I and Model II along with experimental data [27] at T = 200 °C. The O_3_/NO concentrations ranged from 0.0 to 1.1. A similar trend to Figure 4 was obtained.

The simulation results of both Model I and Model II under-predicted the experimental data. Where the O_3_/NO concentration equaled 1, the experiment determined NO concentration as 73 ppm; the simulation results of Model I and Model II were 53 ppm and 35 ppm, respectively.

**Figure 6:**
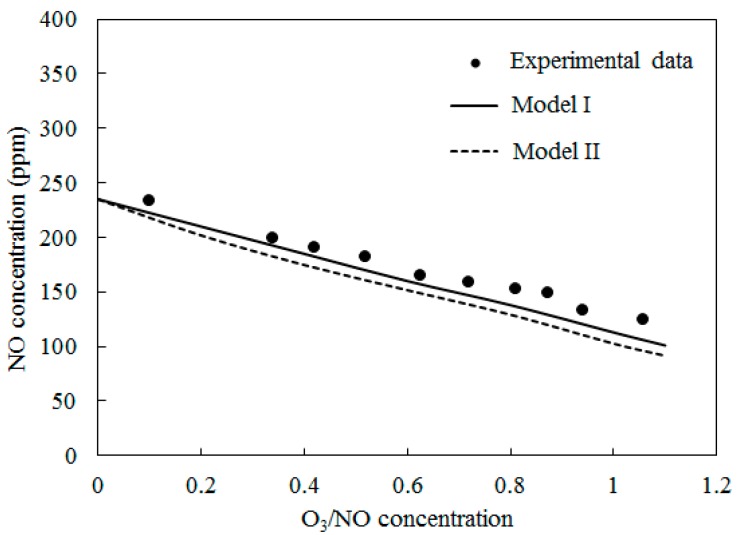
Variations of NO concentration with O_3_/NO concentrations at T = 300 °C.

Figure 6 shows the comparisons of computed NO concentration using Model I and Model II along with experimental data [27] at T = 300 °C. The O_3_/NO concentrations ranged from 0.0 to 1.1. The trends were similar to T = 100 °C and T = 200 °C. The NO concentration decreased as the O_3_/NO concentrations increased. Where the O_3_/NO concentrations equaled 1, the experiment determined the NO concentration as 124 ppm, and simulation results of Model I and Model II were 113 ppm and 93 ppm, respectively. Furthermore, compared with T = 100 °C and T = 200 °C, it was more difficult for NO to react with O_3_ at T = 300 °C.

In Figure 4, Figure 5 and Figure 6, Model I shows closer agreement with experimental data compared with Model II. However, the two models exhibit the same prediction results in Figure 3 at lower temperature (T = 25 °C and T = 40 °C). This result could be attributed to the differences of the reactions in the two models. At higher temperature, reaction O_3_ = O_2_ + O (R4) is more easy to be triggered and provides enough O_2_ for reaction NO + NO + O_2_ = NO_2_ + NO_2_ (R12) is responsible for consuming NO and generating NO_2_, this reaction is included in Model II and it is not included in Model I. Therefore the NO concentration is slightly lower in prediction results of Model II compared with Model I in Figure 4, Figure 5 and Figure 6.

**Figure 7:**
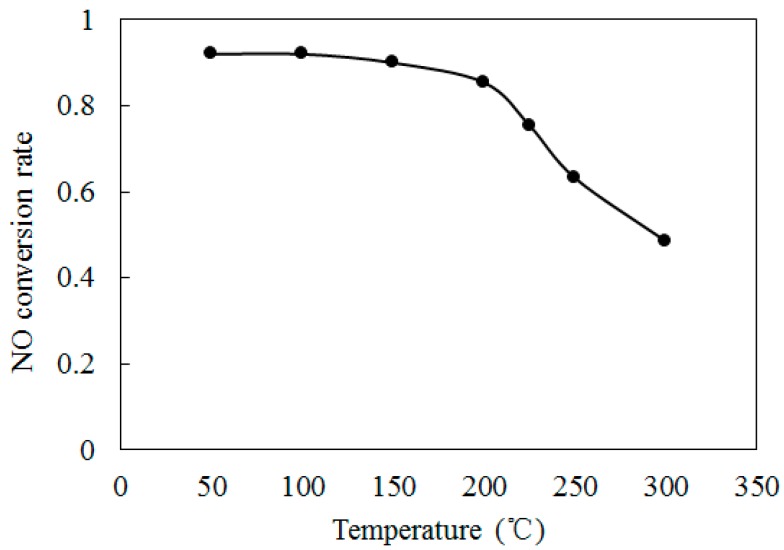
Variations of NO conversion rate with temperatures at O_3_/NO concentration equals 1.0.

To further discuss the effect of initial temperature on the NO conversion, Figure 7 shows the variations of NO conversion rate with temperatures T at O_3_/NO concentration equals 1.0. The initial NO concentration and O_3_ concentration were kept at 300 ppm. The residence time was kept at 1 s. As shown in Figure 7, where temperatures were higher than 200°C, the NO conversion rate was reduced sharply. At T = 300 °C, the NO conversion rate is 48.5%. Previous studies [26,27] demonstrated that O_3_ becomes unstable at higher temperatures and decomposes to O_2._ According to O_3_ = O_2_ + O (R4), NO will react with O through NO + O = NO_2_ (R6), which is more difficult trigger than R1. Therefore, a lesser amount of NO was transformed into NO_2_ at T = 300 °C. The O_3_ decomposition at higher temperature leads to the decrease of NO conversion rate.

**Figure 8:**
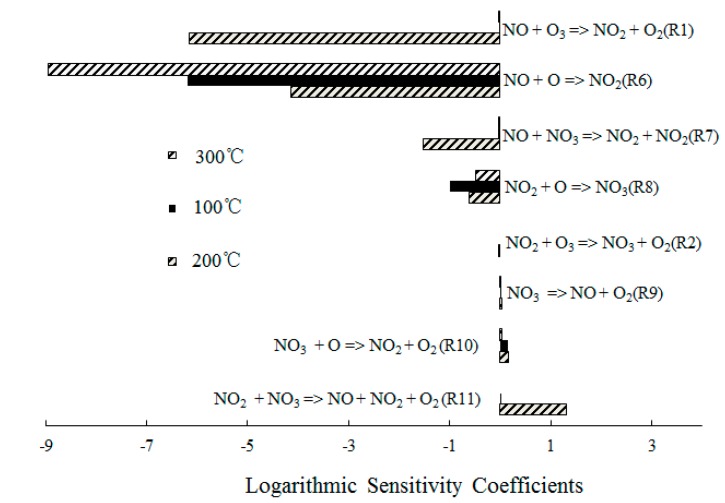
Ranked logarithmic sensitivity coefficients of NO formation where O_3_/NO concentration equals 1.

In order to obtain further insight into the controlling chemical mechanism of the oxidation process, a sensitivity analysis of NO formation and NO_2_ formation was conducted for comparison purposes. Figure 8 depicts the ranked logarithmic sensitivity coefficients of NO formation where the O_3_/NO concentration equaled 1.

Reaction O_3_ + NO = NO_2_ + O_2_ (R1) showed significant results at T = 100 °C, which indicated that the direct oxidation of NO by O_3_ was the main reaction path of NO consumption. However, NO + O = NO_2_ (R6) dominated the oxidation process of NO at T = 300 °C, which was expected for T = 300 °C; The O_3_ decomposed to O_2_ and O, a large amount of O was formed and then reacted with the excess NO. In addition, the reactions NO + NO_3_ = NO_2_ + NO_2_ (R7) and NO_2_ + O = NO_3_ (R8) also showed significant results for the NO consumption. Hence, where a molar ratio of O_3_/NO equaled 1, a small amount of NO_3_ was generated and reacted with NO.

**Figure 9:**
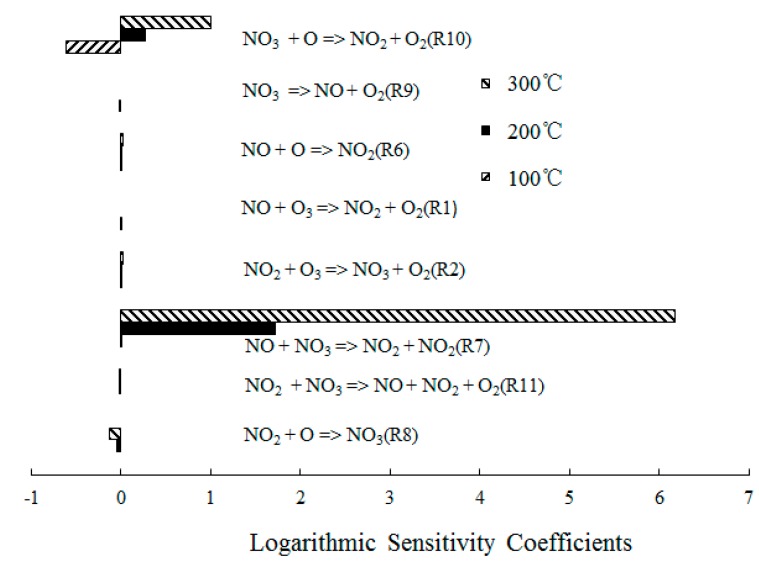
Ranked logarithmic sensitivity coefficients of NO_2_ formation where O_3_/NO concentration equals 1.

Figure 9 depicts the ranked logarithmic sensitivity coefficients of NO_2_ formation where the O_3_/NO concentration equaled 1. NO + NO_3_ = NO_2 _+ NO_2_ (R7) dominated the overall reactivity and also the NO_2_ formation at T = 300 °C. At T = 200 °C, NO + O_3_ = NO_2_ + O_2_ (R1), NO_2_ + O_3_ = NO_3_ + O_2 _(R2), NO + O = NO_2_ (R6) and NO_3_ = NO + O_2 _(R9) had a positive effect on NO_2_ formation. The sensitivity coefficient of NO + O_3_ = NO_2_ + O_2_ (R1) was 0 at T = 300 °C, which confirmed that O_3_ decomposed at T = 300 °C [27]. There was insufficient O_3_ to react with NO; therefore, the reaction NO + O_3_ = NO_2_ + O_2_ (R1) was subsequently difficult to trigger. Furthermore, NO_3_ + O = NO_2_ + O_2_ (R10) showed significant results in the NO_2_ formation due to a large amount of O formation.

Based on the previous discussion, the optimal reaction temperature for O_3_/NO ranged from 100 to 200 °C. As the temperature increased, O_3_ decomposed into O and O_2_ before reacting with NO. The main oxidation reaction NO + O_3_ = NO_2_ + O_2_ (R1) of NO with O_3_ was difficult to trigger; therefore, the NO conversion rates were lower at T = 300 °C compared with T = 100 °C and T = 200 °C. These conclusions indicate that in a coal-fired power plant, the O_3_ injection point should be set before or after the dust removal system, where the temperature of the flue gas is approximately 100~200°C.

**Figure 10:**
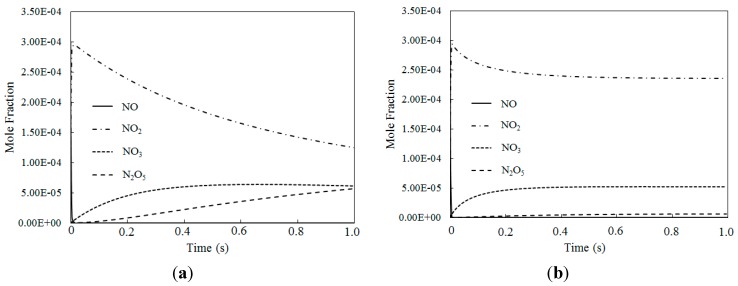
The mole fraction variations for NO, NO_2_, NO_3_ and N_2_O_5 _of different temperatures where O_3_/NO concentrations equal 1.5 for T = 100 °C and T = 200 °C. (**a**) T = 100 °C; (**b**) T = 200 °C.

**Figure 11:**
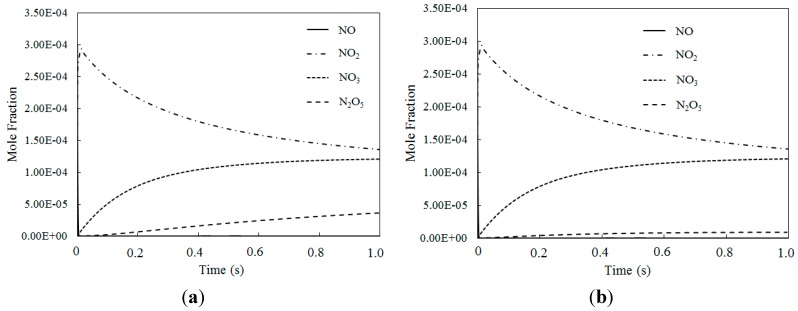
The mole fraction variations of NO, NO_2_, NO_3_ and N_2_O_5_ at different temperatures where O_3_/NO concentration equals 2 for T = 100 °C and T = 200 °C. (**a**) T = 100 °C; (**b**) T = 200 °C.

The above discussions show that when the O_3_/NO concentration was less than 1, most of the NO was oxidized into NO_2_, and a higher oxidation state of NO_2_ was difficult produced due to insufficient O_3_. For a further understanding of possible higher oxidative level NOx species producing in the oxidation process at higher O_3_/NO mole fraction ratios, the molar fraction variations of NO, NO_2_, NO_3_ and N_2_O_5_ for different temperatures where the O_3_/NO concentration equaled 1.5 and 2.0 are discussed in Figure 10 and Figure 11.

Figure 10 depicts the molar fraction variations of NO, NO_2_, NO_3_ and N_2_O_5 _for different temperatures where the O_3_/NO concentration equaled 1.5 (with variable residence time). Figure 10a shows that at T = 100 °C, NO was first consumed, then NO_2_, NO_3_ and N_2_O_5_ were generated. Figure 10b shows similar trends to Figure 10a for T = 200 °C. Compared to an O_3_/NO concentration equal to 1, a higher oxidation state of NO was produced where the O_3_/NO concentration was higher than 1. The reactions R1, R2 and R12 are show significant importance at O_3_/NO concentrations larger than 1.0, which is also confirmed by previous studies [27,33]. At the beginning of the reaction, the NO oxidized to NO_2_ by O_3 _via reaction NO + O_3_ = NO_2_ + O_2_ (R1). As excess O_3_ reacted with NO_2_ via NO_2_ + O_3_ = NO_3_ + O_2 _(R2), NO_2_ rapidly decreased and NO_3_ began to generate. N_2_O_5_ was formed via reaction NO_2_ + NO_3_ = N_2_O_5_ (R12). Hence, NO_3_ and N_2_O_5_ have a higher oxidization state than NO and they can be generated where there is an excess of O_3_. As demonstrated by previous study [25], reaction R2 was much slower than reaction R1, thus the NO_3_ concentration was lower than NO_2_ concentration. In addition, the N_2_O_5_ produced by R12 decomposed into NO_2_ and NO_3_ by its reverse reaction, thus, the N_2_O_5_ concentration was very low compared with NO_2_ and NO_3_. Additionally, compared with T = 100 °C, the NO_3_ mole fractions were higher at T = 200 °C, whereas the N_2_O_5_ mole fractions were lower, this trend was also confirmed by calculated results from Mok* et al.* [25], indicating that at higher temperature, N_2_O_5_ is more easy to be consumed by the reverse reaction of NO_2_ + NO_3_ = N_2_O_5_ (R12).

Figure 11 depicts the mole fraction variations of NO, NO_2_, NO_3_ and N_2_O_5_ for different temperatures where the O_3_/NO concentrations equaled 2.0 (with variable residence time). The NO_2_ mole fraction was nearly identical where the O_3_/NO concentrations equaled 1.5 and 2.0. However, where the O_3_/NO concentrations equaled 2, the NO_3_ mole fraction was higher than when the O_3_/NO concentration equaled 1.5, which indicated that the O_3_/NO concentration had a significant effect on the mole fractions of the nitrogen oxidized products.

Figure 10 and Figure 11 show that when the O_3_/NO concentration was greater than 1, higher oxidized products were formed (such as NO_3_, N_2_O_5_). NO_2_ was first oxidized to produce NO_3_ by the excess of O_3_, and then NO_3_ reacted with NO_2_ to produce N_2_O_5_, obtaining a higher oxidized state of NO.

Simulation results for reaction products where the O_3_/NO concentrations were greater than 1 yielded clear evidence that NO could be oxidized to higher state oxidations such as NO_3_ and N_2_O_5_ by O_3_ with appropriate O_3_/NO concentrations. Additionally, the results confirmed that Model I and Model II could both be used in modelling the experimental data or in predicting the effect of ozone injection into flue gas in practical applications. Finally, to ensure that all NO is transformed into a high oxidation state (such as NO_2_, NO_3_ and N_2_O_5_), molar ratios where O_3_/NO is greater than 1 should be taken into account for practical applications.

### 4.3. SO_2_ Oxidation by O_3_

**Figure 12:**
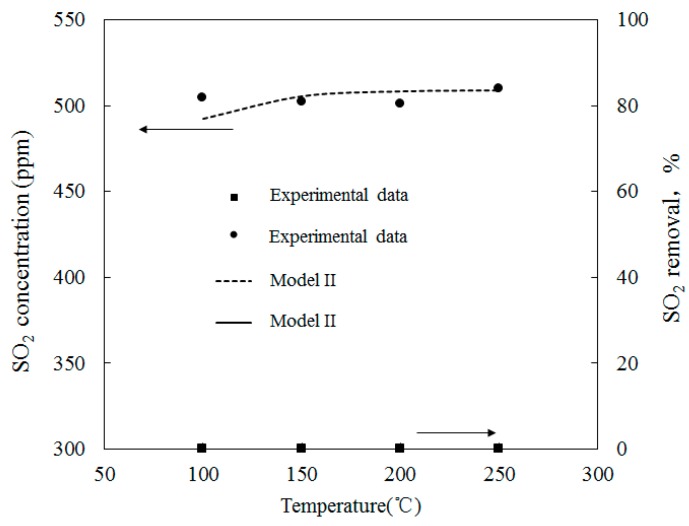
Variations of SO_2_ concentration with temperatures where the mole ratio of O_3_/SO_2_ equals.

Figure 12 depicts the variations of SO_2_ concentrations with temperatures where the molar ratio of O_3_/SO_2_ was equal to 1; SO_2_ reduction rates are also shown. Experimental data provided by Wang [28], along with the simulation results of Model II, are also provided. The initial SO_2_ concentration feeding into the reactor was 500 ppm and the temperature ranged from 100 to 250 °C. Figure 12 also shows that the temperature variations had little effect on the SO_2_ mole concentration.

**Figure 13:**
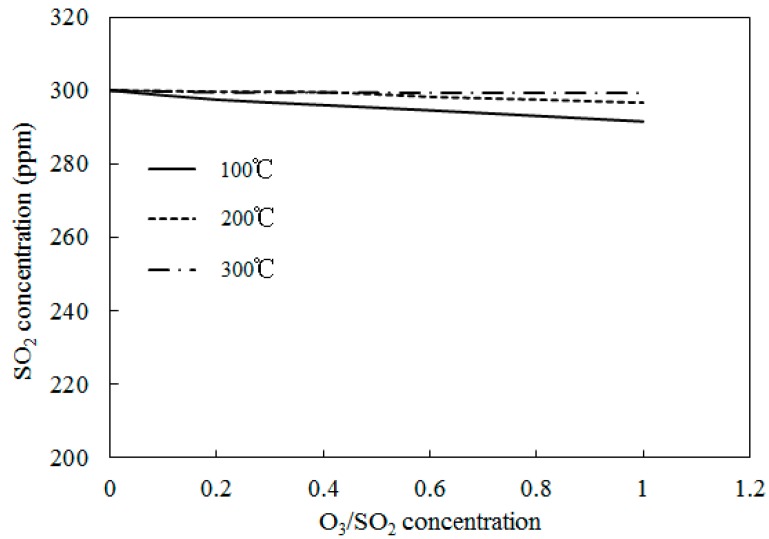
Variations of SO_2_ concentrations with O_3_/SO_2 _mole fractions at different temperatures.

Simulations on the effect of the molar ratio of O_3_/SO_2_ on variations of SO_2_ concentrations were performed in order to obtain further insight into the interactions between O_3_ and SO_2_. Figure 13 depicts the variations of SO_2_ concentrations with O_3_/SO_2_ mole fractions at different temperatures. The O_3_/SO_2_ mole fractions ranged from 0 to 1.0. Simulations were performed with Model II. As discussed in Wang [28], Model I also demonstrated similar trends (not presented in the current study). Figure 12 shows that it was difficult for O_3 _to oxidize SO_2_ at various molar ratios of O_3_/SO_2_. Additionally, at lower temperatures (T = 100 °C), 10 ppm SO_2_ could be transformed into SO_3_ where the mole ratio of O_3_/SO_2_ equaled 1; however, it appeared that the injection of ozone still had little effect on the oxidation of SO_2_. This may be caused by the reaction O_3_ + SO_2_ = O_2_ + SO_3 _(R1)_, _which is the first step for SO_2_ oxidation, had a large energy barrier in both Model I (13,910) and Model II (13,923). As such, it was difficult to trigger this reaction and, therefore, it was difficult for SO_2_ to be oxidized.

For the process of SO_2_ oxidation by O_3_, the results of both experiments and simulations indicated that SO_2_ was not significantly oxidized and that variations of reaction temperatures and O_3_/SO_2_ mole fractions had little effect on the SO_2_ reduction. The appearance of SO_2_ in the flue gas did not consume a large amount of O_3_.

### 4.4. Simultaneous Oxidation of NO and SO_2_ by O_3_

In the flue gas of a coal-fired power plant, large amounts of NO and SO_2_ are mixed; therefore, the interactions between SO_2_ and NO should be taken into account for practical applications of O_3_ injection. Simulations of Model I and Model II, where the O_3_/NO/SO_2_ mole fraction was 2:1:1, were conducted at different temperatures ranging from 100 to 250 °C. The results show that the appearance of NO did not promote the oxidation of SO_2_ by O_3_ and that the conversion of SO_2_ was difficult. Furthermore, the NO conversion rate was not affected by the addition of SO_2_, which shows that SO_2_ had little effect on the oxidation of NO in the simultaneous oxidation of NO and SO_2_ by ozone injection. This conclusion was also confirmed by Sun* et al.* [21] with situ IR measurements. Therefore, with respect to actual applications, O_3_ injections could help NO transform into NO_2_, NO_3_ and N_2_O_5_, which can then be absorbed in the scrubbing tower of the subsequent process with alkaline solution. Almost all of the SO_2_ will be absorbed with the alkaline solution because it is difficult for SO_2_ to be oxidized by the O_3_ injection. Therefore, a scrubbing tower should be applied in order to achieve the simultaneous removal of NO and SO_2_.

## 5. Conclusions

This study used numerical simulations with two kinetic models to investigate the oxidation process of NO, SO_2_ and NO/SO_2_ mixtures by O_3._ The computed results were compared with experimental data from previous research. Two kinetic mechanisms were taken into account for modeling issues.

Both of the models showed satisfactory agreement with the experimental data with regards to NO oxidization by O_3_. The results showed that NO concentration decreased as the mole ratio of O_3_/NO increased (where the mole ratio of O_3_/NO was less than 1). NO_2_ was the main product of the NO oxidation. The optimal reaction temperature for O_3_/NO ranged from 100 to 200 °C. As the temperature increased, O_3_ easily decomposed to O and O_2_ before reacting with NO. As such, the main oxidation reaction R1 of NO with O_3_ was difficult to trigger. Therefore, the NO conversion rates were lower at T = 300 °C compared with T = 100 °C and T = 200 °C.

When the molar ratio of O_3_/NO was greater than 1, a higher oxidation state of NO (such as NO_3_ and N_2_O_5)_ was formed. Reaction NO_3_ + O_3_ = NO_2_ + O_2_ and reaction NO_2_ + NO_3_ = N_2_O_5_ showed significant results in the oxidation process. First, NO_2_ was generated; NO_3_ was then produced by the oxidization of NO_2_ and finally N_2_O_5_ was produced by NO_3_ reacting with NO_2_. Therefore, for practical applications, mole ratios of O_3_/NO greater than 1 are recommended in order to ensure that all NO is transformed into a high oxidation state (such as NO_2_, NO_3_ and N_2_O_5_).

Both the experimental results and numerical simulation results showed that the oxidation of SO_2_ was unaffected by injected ozone at different temperatures and mole ratios of O_3_/SO_2_. Computations of NO/SO_2_/O_3_ showed that the NO conservation rate was not affected by the addition of SO_2_ and that SO_2_ had little effect on the oxidation of NO in the simultaneous oxidation of NO and SO_2_ by the ozone injection. In summary, this study used two kinetic models for modeling the oxidation process of NO and SO_2_ by O_3_ injections. The optimized temperatures and mole ratios for NO/O_3_ for further applications of O_3_ injection for the control of pollutant emission in coal-fired power plants were suggested. Based on this study, a potentially viable facility for the simultaneous removal of NO, SO_2_ and PM_2.5 _was proposed.

## Supplementary Files

Supplementary File 1
