# Supplementary material for: Numerical Study of the Simultaneous Oxidation of NO and SO2 by Ozone
Source: Int J Environ Res Public Health. 2015 Jan 29;12(2):1595–611. doi: 10.3390/ijerph120201595 (PMC4344682; doi:10.3390/ijerph120201595)
Supplement: Supplementary File 1 [file ijerph-12-01595-s001.pdf]

# Numerical Study of Simultaneous Oxidation of NO and $SO_2$ by Ozone

## Model I

| Reaction | A ($cm^3$/mol-s) | n | Ea (cal/mol) |
|---|---|---|---|
| $O_3 + H = O_2 + OH$ | 1.64E + 13 | 0.75 | 750 |
| $O_3 + H = O + HO_2$ | 4.52E + 11 | 0 | 0 |
| $O_3 + OH = O_2 + HO_2$ | 1.15E + 12 | 0 | 1990 |
| $O_3 + H_2O = O_2 + H_2O_2$ | 6.62E + 10 | 0 | 0 |
| $O_3 + HO_2 = 2O_2 + OH$ | 1.19E + 08 | 4.57 | 1380 |
| $O_3 + N = O_2 + NO$ | 6.00E + 07 | 0 | 0 |
| $O_3 + NO = NO_2 + O_2$ | 1.80E + 12 | 0 | 2722 |
| $O_3 + NO_2 = O_2 + NO_3$ | 7.22E + 10 | 0 | 4870 |
| $O_3 = O_2 + O$ | 2.00E + 15 | 0 | 23,250 |
| $O_3 + O = 2O_2$ | 4.82E + 12 | 0 | 4093 |
| $H + O_2 + M = HO_2 + M$ | 3.61E + 17 | −0.72 | 0 |
| $H + H + M = H_2 + M$ | 1.00E + 18 | −1 | 0 |
| $H + H + H_2 = H_2 + H_2$ | 9.20E + 16 | −0.6 | 0 |
| $H + H + H_2O = H_2 + H_2O$ | 6.00E + 19 | −1.25 | 0 |
| $H + OH + M = H_2O + M$ | 1.60E + 22 | −2 | 0 |
| $H + O + M = OH + M$ | 6.20E + 16 | −0.6 | 0 |
| $O + O + M = O_2 + M$ | 1.89E + 13 | 0 | −1788 |
| $H_2O_2 + M = OH + OH + M$ | 1.30E + 17 | 0 | 45,500 |
| $H_2 + O_2 = 2OH$ | 1.70E + 13 | 0 | 47,780 |
| $OH + H_2 = H_2O + H$ | 1.17E + 09 | 0 | 3626 |
| $O + OH = O_2 + H$ | 3.61E + 14 | −0.5 | 0 |
| $O + H_2 = OH + H$ | 5.06E + 04 | 2.67 | 6290 |
| $O + HO_2 = O_2 + OH$ | 1.40E + 13 | 0 | 1073 |
| $_2OH = O + H_2O$ | 6.00E + 08 | 1.3 | 0 |
| $H + HO_2 = H_2 + O_2$ | 1.25E + 13 | 0 | 0 |
| $H_2O_2 + H = HO_2 + H_2$ | 1.60E + 12 | 0 | 3800 |
| $N + O_2 = NO + O$ | 6.40E + 09 | 1 | 6280 |
| $N + OH = NO + H$ | 3.80E + 13 | 0 | 0 |
| $NO + M = N + O + M$ | 9.64E + 14 | 0 | 620,910 |
| $N + HO_2 = NO + OH$ | 1.00E + 13 | 0 | 8390 |
| $NO + N = N_2 + O$ | 3.27E + 12 | 0.3 | 0 |
| $NO + O + M = NO_2 + M$ | 1.30E + 15 | −0.75 | 0 |
| $NO + OH = HNO_2$ | 5.45E + 17 | 0 | 0 |
| $HNO_3 + O = NO_3 + OH$ | 1.81E + 07 | 0 | 0 |
| $HNO_3 + H = NO_3 + H_2$ | 3.40E + 12 | 1.53 | 16,332 |
| $HNO_3 + H = NO_2 + H_2O$ | 8.39E + 09 | 3.29 | 6255 |
| $HNO_3 + NO = NO_2 + HNO_2$ | 4.48E + 03 | 0 | 0 |
| $HNO_3 + OH = NO_3 + H_2O$ | 4.82E + 08 | 0 | 0 |
| $HNO_3 + OH = NO_2 + H_2O_2$ | 4.82E + 08 | 0 | 0 |
| $HNO_3 = NO_2 + OH$ | 6.90E + 17 | 0 | 45,730 |
| $HNO + O = NO + OH$ | 2.29E + 13 | 0 | 0 |
| $HNO + HNO = N_2O + H_2O$ | 2.55E + 07 | 3.98 | 1188 |

| | | | |
|---|---|---|---|
| $HNO + H = NO + H_2$ | 2.70E + 13 | 0.72 | 651 |
| $HNO + NO_2 = NO + HNO_2$ | 6.03E + 11 | 0 | 1980 |
| $HNO + OH = NO + H_2O$ | 4.82E + 13 | 0 | 990 |
| $H + HO_2 = OH + OH$ | 8.22E + 12 | 0.75 | 0 |
| $NO_2 + HO_2 = HNO_2 + O_2$ | 2.20E−01 | 0 | 0 |
| $NO + HO_2 = HNO + O_2$ | 5.84E + 05 | 0 | 5600 |
| $NO + HO_2 = NO_2 + OH$ | 6.32E + 11 | 0.58 | 1430 |
| $NO + HO_2 = HNO_3$ | 3.47E + 12 | 0 | −5720 |
| $H_2O + HO_2 = H_2O_2 + OH$ | 2.80E + 13 | 0 | 32,790 |
| $H_2O_2 + HO_2 = O_2 + H_2O + OH$ | 6.03E + 10 | 0 | 0 |
| $OH + HO_2 = O_2 + H_2O$ | 4.28E + 13 | −0.21 | 110 |
| $HO_2 + HO_2 = H_2O_2 + O_2$ | 1.87E + 12 | 0 | 1540 |
| $HO_2 = H + O_2$ | 1.45E + 16 | −1.18 | 48,490 |
| $NO_2 + NO_3 = N_2O_5$ | 7.98E + 17 | −3.9 | 0 |
| $N + NO_2 = O + O + N_2$ | 1.30E−01 | 0 | 0 |
| $NO_2 + H = NO + OH$ | 2.41E + 14 | 0 | 680 |
| $NO + NO = O_2 + N_2$ | 3.10E + 13 | 0 | 63,190 |
| $NO + N_2O = NO_2 + N_2$ | 1.73E + 11 | 2.23 | 46,300 |
| $H_2S + M = S + H_2 + M$ | 1.60E + 24 | −2.61 | 44,800 |
| $H_2S + H = SH + H_2$ | 1.20E + 07 | 2.1 | 350 |
| $H_2S + O = SH + OH$ | 7.50E + 07 | 1.75 | 1460 |
| $H_2S + OH = SH + H_2O$ | 2.70E + 12 | 0 | 0 |
| $H_2S + S = 2SH$ | 8.30E + 13 | 0 | 3700 |
| $H_2S + S = HS_2 + H$ | 2.00E + 13 | 0 | 3723.8 |
| $S + H_2 = SH + H$ | 1.40E + 14 | 0 | 9700 |
| $SH + O = H + SO$ | 1.00E + 14 | 0 | 0 |
| $SH + OH = S + H_2O$ | 1.00E + 13 | 0 | 0 |
| $SH + HO_2 = HSO + OH$ | 1.00E + 12 | 0 | 0 |
| $SH + O_2 = HSO + O$ | 1.90E + 13 | 0 | 9000 |
| $S + OH = H + SO$ | 4.00E + 13 | 0 | 0 |
| $S + O_2 = SO + O$ | 5.20E + 06 | 1.81 | −600 |
| $_2SH = S_2 + H_2$ | 1.00E + 12 | 0 | 0 |
| $SH + S = S_2 + H$ | 1.00E + 13 | 0 | 0 |
| $S_2 + M = {}_2S + M$ | 4.80E + 13 | 0 | 38,800 |
| $S_2 + H + M = HS_2 + M$ | 1.00E + 16 | 0 | 0 |
| $S_2 + O = SO + S$ | 1.00E + 13 | 0 | 0 |
| $HS_2 + H = S_2 + H_2$ | 1.20E + 07 | 2.1 | 352.42 |
| $HS_2 + O = S_2 + OH$ | 7.50E + 07 | 1.8 | 1460 |
| $HS_2 + OH = S_2 + H_2O$ | 2.70E + 12 | 0 | 0 |
| $HS_2 + S = S_2 + SH$ | 8.30E + 13 | 0 | 3700 |
| $HS_2 + H + M = H_2S_2 + M$ | 1.00E + 16 | 0 | 0 |
| $N_2/1.5/SO_2/10/H_2O$ | | | |
| $H_2S_2 + H = HS_2 + H_2$ | 1.20E + 07 | 2.1 | 360 |
| $H_2S_2 + O = HS_2 + OH$ | 7.50E + 07 | 1.8 | 1460 |
| $H_2S_2 + OH = HS_2 + H_2O$ | 2.70E + 12 | 0 | 0 |
| $H_2S_2 + S = HS_2 + SH$ | 8.30E + 13 | 0 | 3700 |
| $SO_3 + H = HOSO + O$ | 2.50E + 05 | 2.92 | 25,300 |
| $SO_3 + O = SO_2 + O_2$ | 2.00E + 12 | 0 | 10,000 |
| $SO_3 + SO = 2SO_2$ | 1.00E + 12 | 0 | 5000 |
| $SO + O (+M) = SO_2( + M)$ | 3.20E + 13 | 0 | 0 |

| | | | |
|---|---|---|---|
| $SO_2$ + O (+M) = $SO_3$ (+M) | 9.20E + 10 | 0 | 1200 |
| $SO_2$ + OH (+M) = $HOSO_2$ (+M) | 5.73E + 12 | −0.27 | 0 |
| $SO_2$ + OH = HOSO + O | 3.90E + 08 | 1.89 | 38,200 |
| $SO_2$ + OH = $SO_3$ + H | 4.90E + 02 | 2.69 | 12,000 |
| $SO_2$ + CO = SO + $CO_2$ | 2.70E + 12 | 0 | 24,300 |
| SO + M = S + O + M | 4.00E + 14 | 0 | 54,000 |
| SO + H + M = HSO + M | 5.00E + 15 | 0 | 0 |
| HOSO (+M) = SO + OH (+M) | 9.94E + 21 | −2.54 | 38,190 |
| SO + OH = $SO_2$ + H | 1.077E + 17 | −1.35 | 0 |
| SO + $O_2$ = $SO_2$ + O | 7.60E + 03 | 2.37 | 1500 |
| 2SO = $SO_2$ + S | 2.00E + 12 | 0 | 2000 |
| HSO + H = HSOH | 2.50E + 20 | −3.14 | 460 |
| HSO + H = SH + OH | 4.90E + 19 | −1.86 | 785 |
| HSO + H = S + $H_2O$ | 1.60E + 09 | 1.37 | −170 |
| HSO + H = $H_2SO$ | 1.80E + 17 | −2.47 | 25 |
| HSO + H = $H_2S$ + O | 1.10E + 06 | 1.03 | 5230 |
| HSO + H = SO + $H_2$ | 1.00E + 13 | 0 | 0 |
| HSO + O + M = $HSO_2$ + M | 1.10E + 19 | −1.73 | −25 |
| HSO + O = $SO_2$ + H | 4.50E + 14 | −0.4 | 0 |
| HSO + O + M = HOSO + M | 6.90E + 19 | −1.61 | 800 |
| HSO + O = O + HOS | 4.80E + 08 | 1.02 | 2700 |
| HSO + O = OH + SO | 1.40E + 13 | 0.15 | 150 |
| HSO + OH = HOSHO | 5.20E + 28 | −5.44 | 1600 |
| HSO + OH = HOSO + H | 5.30E + 07 | 1.57 | 1900 |
| HSO + OH = SO + $H_2O$ | 1.70E + 09 | 1.03 | 235 |
| HSO + $O_2$ = $SO_2$ + OH | 1.00E + 12 | 0 | 5000 |
| HSOH = SH + OH | 2.80E + 39 | −8.75 | 37,800 |
| HSOH = S + $H_2O$ | 5.80E + 29 | −5.6 | 27,400 |
| HSOH = $H_2S$ + O | 9.80E + 16 | −3.4 | 43,500 |
| $H_2SO$ = $H_2S$ + O | 4.90E + 28 | −6.66 | 36,000 |
| H + $SO_2$ (+M) = HOSO (+M） | 3.12E + 08 | 1.61 | 3606 |
| HOSO + M = O + HOS + M | 2.50E + 30 | −4.8 | 60,000 |
| HOSO + H = $SO_2$ + $H_2$ | 3.00E + 13 | 0 | 0 |
| HOSO + H = SO + $H_2O$ | 6.30E−10 | 6.29 | −960 |
| HOSO + OH = $SO_2$ + $H_2O$ | 1.00E + 12 | 0 | 0 |
| HOSO + $O_2$ = $HO_2$ + $SO_2$ | 1.00E + 12 | 0 | 500 |
| $HSO_2$ + H = $SO_2$ + $H_2$ | 3.00E + 13 | 0 | 0 |
| $HSO_2$ + OH = $SO_2$ + $H_2O$ | 1.00E + 13 | 0 | 0 |
| $HSO_2$ + $O_2$ = $HO_2$ + $SO_2$ | 1.00E + 13 | 0 | 0 |
| H + $SO_2$ (+M) = $HSO_2$ (+M) | 1.06E + 09 | 1.48 | 594.6 |
| $HOSO_2$ = HOSO + O | 5.40E + 18 | −2.34 | 53,500 |
| $HOSO_2$ = $SO_3$ + H | 1.40E + 18 | −2.91 | 27,600 |
| $HOSO_2$ + H = $SO_2$ + $H_2O$ | 1.00E + 12 | 0 | 0 |
| $HOSO_2$ + O = $SO_3$ + OH | 5.00E + 12 | 0 | 0 |
| $HOSO_2$ + OH = $SO_3$ + $H_2O$ | 1.00E + 12 | 0 | 0 |
| $HOSO_2$ + $O_2$ = $HO_2$ + $SO_3$ | 7.80E + 11 | 0 | 330 |
| HOSHO = HOSO + H | 6.40E + 30 | −5.89 | 37,100 |
| HOSHO = SO + $H_2O$ | 1.20E + 24 | −3.59 | 30,000 |
| HOSHO + H = HOSO + $H_2$ | 1.00E + 12 | 0 | 0 |
| HOSHO + O = HOSO + OH | 5.00E + 12 | 0 | 0 |

| | | | |
|---|---|---|---|
| $HOSHO + OH = HOSO + H_2O$ | 1.00E + 12 | 0 | 0 |
| $C + SO_2 = CO + SO$ | 4.156E + 13 | 0.00 | 0 |
| $HOSO_2 + H = SO_3 + H_2$ | 1.00E + 12 | 0 | 0 |
| $S + CH4 = SH + CH_3$ | 6.00E + 14 | 0 | 1278.42 |
| $H_2S + CH_3 = CH4 + SH$ | 1.80E + 11 | 0 | 1177.53 |
| $SH + O = S + OH$ | 1.00E + 14 | 0 | 0 |
| $C + H_2S = CH + SH$ | 1.20E + 14 | 4450 | 0.32 |
| $O + COS = CO + SO$ | 1.93E + 13 | 232 | 8.6 |
| $O + CS = CO + S$ | 1.63E + 14 | 0 | 760.16 |
| $COS + M = CO + S + M$ | 1.43E + 14 | 307 | 0.02 |
| $O + COS = CO_2 + S$ | 5.00E + 13 | 0 | 5530.4 |
| $SH + O_2 = SO + OH$ | 1.00E + 12 | 503 | 2.48 |
| $CH + SO = CO + SH$ | 1.00E + 13 | 0 | 0 |
| $SO_3 + S = SO + SO_2$ | 5.12E + 11 | 0 | 0 |
| $SH + NO = SN + OH$ | 1.00E + 13 | 0 | 8900.7 |
| $S + NO = SN + O$ | 1.00E + 12 | 0.5 | 17,501 |
| $SH + NH = SN + H_2$ | 1.00E + 14 | 0 | 0 |
| $N + SO = NO + S$ | 6.31E + 11 | 0.5 | 1010.3 |
| $N + SH = SN + H$ | 6.31E + 11 | 0.5 | 4030.6 |
| $SN + NO = N_2 + SO$ | 1.81E + 10 | 0 | 0 |
| $SN + O_2 = SO + NO$ | 3.00E + 08 | 0 | 0 |
| $SN + NO_2 = S + NO + NO$ | 8.00E + 15 | −0.98 | 0 |
| $N + SN = N_2 + S$ | 6.30E + 11 | 0.5 | 0 |
| $SO_2 + NO_2 = NO + SO_3$ | 4.25E−19 | 8.9 | 3797.21 |
| $SO + NO_2 = SO_2 + NO$ | 8.43E + 12 | 0 | 0 |
| $SN + O = SO + N$ | 6.31E + 11 | 0.5 | 4030.6 |
| $S + NH = SH + N$ | 1.00E + 13 | 0 | 0 |
| $NH + SO = NO + SH$ | 3.01E + 13 | 0 | 0 |
| $HSO + NO_2 = HOSO + NO$ | 5.80E + 12 | 0 | 0 |
| $SO_2 + O_3 = O_2 + SO_3$ | 1.81E + 12 | 0 | 13,910 |
| $SO_3 + H_2O = H_2SO_4$ | 7.23E + 08 | 0 | 0 |

## Model II

| Reaction | A ($cm^3$/mol-s) | n | Ea (cal/mol) |
|---|---|---|---|
| $O_3 \geq O_2 + O$ | 4.31E + 14 | 0 | 22,277 |
| $O_3 + O \geq O_2 + O_2$ | 4.82E + 12 | 0 | 4098 |
| $O + O \geq O_2$ | 1.89E + 13 | 0 | −1789 |
| $H_2O + O_3 \geq H_2O_2 + O_2$ | 66.2 | 0 | 0 |
| $H_2O_2 + O \geq HO_2 + OH$ | 8.43E + 11 | 0 | 3978 |
| $H_2O_2 + OH \geq HO_2 + H_2O$ | 1.75E + 12 | 0 | 318 |
| $H_2O + O \geq OH + OH$ | 7.53E + 12 | 1.3 | 17,105 |
| $HO_2 + O \geq OH + O_2$ | 1.63E + 13 | 0 | −444 |
| $HO_2 + O_3 \geq OH + O_2 + O_2$ | 1.19E + 08 | 4.57 | −1377 |
| $HO_2 + OH \geq H_2O + O_2$ | 2.89E + 13 | 0 | −497 |
| $HO_2 + H_2O \geq OH + H_2O_2$ | 2.80E + 13 | 0 | 32,775 |
| $HO_2 + H_2O_2 \geq OH + H_2O + O_2$ | 6.03E + 10 | 0 | 0 |
| $HO_2 + HO_2 \geq H_2O_2 + O_2$ | 6.17E + 14 | 0 | −1988 |
| $OH + OH \geq H_2O + O$ | 3.73E + 10 | 2.6 | −1880 |
| $H_2O_2 \geq OH + OH$ | 1.22E + 21 | −4.86 | 53,349 |
| $OH + O_3 \geq HO_2 + O_2$ | 1.02E + 12 | 0 | 1870 |
| $NO + O \geq NO_2$ | 1.80E + 13 | 0.3 | 0 |

| | | | |
|---|---|---|---|
| $NO_2 + O \geq NO_3$ | 1.38E + 13 | 0.24 | 0 |
| $NO_3 + O \geq O_2 + NO_2$ | 1.02E + 13 | 0 | 0 |
| $NO + NO + O_2 \geq NO_2 + NO_2$ | 1.20E + 09 | 0 | −1055 |
| $NO + O_3 \geq NO_2 + O_2$ | 8.43E + 11 | 0 | 2605 |
| $NO_2 + O_3 \geq NO_3 + O_2$ | 8.43E + 10 | 0 | 4913 |
| $NO_2 + NO_3 \geq N_2O5$ | 1.14E + 12 | 0.2 | 0 |
| $NO + OH \geq HNO_2$ | 1.99E + 13 | −0.3 | 0 |
| $NO_2 + OH \geq HNO_3$ | 9.06E + 17 | −4.4 | 0 |
| $NO_2 + OH \geq HO_2 + NO$ | 1.81E + 13 | 0 | 6684 |
| $NO_3 + OH \geq HO_2 + NO_2$ | 1.20E + 13 | 0 | 0 |
| $NO + H_2O_2 \geq H_2O + NO_2$ | 4.22E + 04 | 0 | 0 |
| $NO + H_2O_2 \geq OH + HNO_2$ | 3.13E + 04 | 0 | 0 |
| $NO_3 + H_2O_2 \geq HO_2 + HNO_3$ | 1.21E + 09 | 0 | 0 |
| $NO + HO_2 \geq OH + NO_2$ | 2.17E + 12 | 0 | −535 |
| $NO + HO_2 \geq HNO_3$ | 3.85E + 07 | 0 | −3270 |
| $NO + HO_2 \geq HNO_2 + O$ | 3.01E + 08 | 0 | 0 |
| $NO_3 + HO_2 \geq HNO_3 + O_2$ | 1.15E + 12 | 0 | 0 |
| $NO_3 + HO_2 \geq OH + O_2 + NO_2$ | 1.51E + 12 | 0 | 0 |
| $H_2O + NO + NO_2 \geq HNO_2 + HNO_2$ | 1.60E + 08 | 0 | 0 |
| $H_2O + N_2O5 \geq HNO_3 + HNO_3$ | 1.51E + 02 | 0 | 0 |
| $NO_2 + NO_2 \geq N_2O4$ | 6.02E + 11 | 0 | 0 |
| $N_2O4 \geq NO_2 + NO_2$ | 1.15E + 16 | 0 | 12,849 |
| $H_2O + N_2O4 \geq HNO_2 + HNO_3$ | 2.52E + 14 | 0 | 11,595 |
| $HNO_2 + OH \geq NO_2 + H_2O$ | 1.51E + 12 | 0 | −516 |
| $HNO_2 + NO_3 \geq HNO_3 + NO_2$ | 1.21E + 09 | 0 | 0 |
| $HNO_2 + O_3 \geq HNO_3 + O_2$ | 3.01E + 05 | 0 | 0 |
| $HNO_3 + OH \geq NO_3 + H_2O$ | 9.04E + 10 | 0 | 0 |
| $HNO_3 + O \geq NO_3 + OH$ | 1.81E + 07 | 0 | 0 |
| $NO_3 \geq NO + O_2$ | 2.50E + 06 | 0 | 12,133 |
| $NO_3 + NO_3 \geq NO_2 + NO_2 + O_2$ | 5.12E + 11 | 0 | 4873 |
| $NO_2 + NO_3 \geq NO + NO_2 + O_2$ | 2.71E + 10 | 0 | 2507 |
| $N_2O5 \geq NO_2 + NO_3$ | 9.69E + 14 | 0.1 | 0 |
| $NO + NO_3 \geq NO_2 + NO_2$ | 1.08E + 13 | 0 | −217 |
| $HNO_2 \geq OH + NO$ | 1.19E + 21 | −3.8 | 50,239 |
| $HNO_2 + HNO_2 \geq H_2O + NO + NO_2$ | 6.03E + 03 | 0 | 0 |
| $HNO_3 + NO \geq HNO_2 + NO_2$ | 4.48E + 03 | 0 | 0 |
| $HNO_3 \geq OH + NO_2$ | 6.90E + 17 | 0 | 45,933 |
| $HNO_3 \geq HO_2 + NO$ | 4.76E + 22 | −6.55 | 51,913 |
| $SO_2 + O \geq SO_3$ | 9.20E + 10 | 0 | 1200 |
| $SO_2 + HO_2 \geq OH + SO_3$ | 5.20E + 08 | 0 | 0 |
| $SO_2 + O_3 \geq SO_3 + O_2$ | 1.81E + 12 | 0 | 13,923 |
| $SO_2 + OH \geq HSO_3$ | 1.09E + 17 | −3.3 | 0 |
| $HSO_3 \geq SO_2 + OH$ | 1.90E + 20 | −4.72 | 27,033 |
| $SO_2 + NO_2 \geq SO_3 + NO$ | 0.01 | 0 | 0 |
| $SO_2 + NO_3 \geq SO_3 + NO_2$ | 6.02E + 04 | 0 | 0 |
| $SO_2 + N_2O5 \geq SO_3 + N_2O_4$ | 5.48 | 0 | 0 |

© 2015 by the authors; licensee MDPI, Basel, Switzerland. This article is an open access article distributed under the terms and conditions of the Creative Commons Attribution license (http://creativecommons.org/licenses/by/4.0/).
